# An improved high-resolution method for quantitative separation of empty and filled AAV8 capsids by strong anion exchange HPLC

**DOI:** 10.3389/fbioe.2024.1436857

**Published:** 2024-10-01

**Authors:** Samantha Schrecke, Kevin McManus, Cassandra Moshfegh, Jessica Stone, Thuy-Uyen Nguyen, Gustavo Rivas, Ismaeel Muhamed, Daniel A. J. Mitchell

**Affiliations:** Matica Biotechnology, Inc., Analytical Development Department, Bryan, TX, United States

**Keywords:** aav, rAAV, empty capsids, filled capsids, strong anion exchange, HPLC, gene therapy, CGT

## Abstract

Cell and gene therapy (CGT) is a field of therapeutic medicine that aims to treat, prevent, and cure diseases using engineered cells (stem cells, immune cells, and differentiated adult or fetal cells), vectors [Adeno Associated Virus (AAV), Adeno Virus (AV), Herpes Simplex Virus (HSV), Baculo Virus (BV), Lenti Virus (LV), Retro Virus (RV), etc.], and other carriers [non-viral vectors, virus-like particles (VLP), Lipid Nano-Particles (LNP), etc.]. Among viral CGT vectors, adeno-associated viruses and lentiviruses (AAV and LV) are the most widely applied vector platforms. The presence of non-functional (empty or non-infectious) vectors that carry null or partial genes in the final drug product is classified as an impurity by the FDA. These impurities impair dosage accuracy and induce non-specific immunogenicity and variability in drug efficacy. These non-functional viral vectors in the drug product need to be elucidated following International Conference on Harmonization (ICH) guidelines for clinical manufacturing of the final drug product. This article showcases an ion-exchange chromatography (IEX) high-resolution method supporting ICH guidelines using commercially available AAV8 filled and empty capsids as reference standards. Our method successfully separated empty to full capsids with a resolution of 15 and sustained a linearity greater than 0.98 even under a wide range of empty or full viral particle concentrations (E+9 to E+13 vp/mL), which is an upgrade to other IEX capsid separation methods. The medium-throughput capacity and shorter sample processing time improve testing efficiency and save costs while delivering quality as value. The discussed method is a reliable and reproducible platform to precisely evaluate the presence of non-functional viral particles in AAV8 samples. Aligned with other orthogonal results, the method is a powerful tool to improve the quality of rAAV analytics.

## 1 Introduction

The field of gene therapy drugs (siRNA, shRNA, CRISPR/CAS9, antisense oligos, miRNA, mRNA, viral vaccines, and cellular agents) holds immense promise to treat genetic disorders by delivering genes, agents, and vectors that target specific tissues and cells to support gain-of-function or loss-of-function or revert to a defective phenotype (Pan et al., 2021). To achieve effective gene delivery, viral vectors are commonly used as vehicles and carriers, and among them, AAV-based drugs have gained traction in recent years, and more than 200 drugs are in clinical trials in 2023 (Zhao et al., 2022; Lundstrom, 2023).

AAVs are nonenveloped ssDNA viruses that belong to the family *Parvoviridae*, genus *Dependoparvovirus*. The AAV capsid has the structure of an icosahedron made of 60 monomers composed of viral proteins (VP1, VP2, and VP3) that package a ∼5-kb genome (Nam et al., 2007; Bennett et al., 2017). They require a co-infecting helper virus (e.g., an adenovirus or a herpesvirus) to infect and replicate inside host cells at high efficiencies (Meier et al., 2020). In gene therapy applications, AAVs express transgenes in episomal form to limit or revert a defective phenotype in target cells (Penaud-Budloo et al., 2008; Berns and Muzyczka, 2017; Su et al., 2023). The cost of gene therapy products relies on the process and product efficiency (the cost to manufacture), yield per batch of production, and clinical application. AAV yield per cell is dependent upon i) plasmid or helper co-infection and internalization, ii) rate of AAV-related protein and DNA synthesis, iii) their packaging efficiency, and iv) assembly inside the infected cell (Wang et al., 2015; Bennett et al., 2017). The effectiveness of AAV genomic DNA packaged inside the assembled AAV capsid strongly influences the ratio of filled to empty capsids (Mietzsch et al., 2021). Partially filled AAV capsids arise when incomplete AAV genomes are packaged into intact capsids (Bennett et al., 2017). In gene therapy, these partial AAVs carry a portion of the viral genome and are expected to be infectious. Experimentally, they have a lower GOI expression than filled AAV capsids and tremendously reduce drug efficacy (Troxell et al., 2023a). Other host cell DNA fragments and plasmids may also be packaged along with AAV genomes but at a lower probability, as they do not carry inverted terminal repeats (ITRs) or packaging motifs (Bennett et al., 2017; Asaad et al., 2023).

To increase viral vector safety, custom recombinant AAV (rAAV) vectors are generated from engineered plasmids without the self-replication ability, thereby drastically reducing the possibility of generating replication-competent progeny particles. By engineering AAV’s external capsid structures, the rAAV’s tissue tropism, transduction efficacy, and antigenicity are modified (Pupo et al., 2022). The rAAV genome is divided and split to be carried in different plasmids. When separate plasmids carry a single copy of *rep*, *cap*, and transgene-*ITR*, the probability of all three genes being packaged together by recombination events is greatly reduced (Sena-Esteves and Gao, 2020). However, even when high AAV production is achieved, it frequently produces a considerable amount of empty and or partially filled capsids that should be separated from the full capsids before being considered a final product during manufacturing. The purity of the final product is a paramount factor that could mitigate the adverse effects of rAAV in gene therapy (Sherafat and AC Planning Working Group, 2021; BioPhorum, 2022; McColl-Carboni et al., 2024). By reducing the percentage of empty and partial AAV capsids and controlling AAV dosage, the non-intended inflammatory responses are reduced (Mingozzi and High, 2013). Current tools such as AUC, charged detection mass spectrometry (CDMS), cIEF, TEM, and SEC-MALS are capable of separating and or quantifying the percentage of empty-to-filled capsids (Burnham et al., 2015; Cole et al., 2021; Strasser et al., 2021; Troxell et al., 2023b; McColl-Carboni et al., 2024). However, they require relatively pure samples, higher concentration, have limited throughput with operation backlogs, and require skilled staff for QC tech transfer and qualification.

Strong ion exchange chromatography (IEX) can quantify the full vs. empty capsid ratio in final AAV products (Aebischer et al., 2022; Joshi et al., 2022). However, the existing IEX methods have reduced resolution (1.5 to 7), which is not sufficient to clearly test in-process samples (Gagnon et al., 2021; Khatwani et al., 2021; Khanal et al., 2023; Kurth et al., 2024). Our method has a resolution of up to 15 and can uniquely identify the retention time (Rt) signature of AAV8 empty virus-like particles (VLPs) and AAV8 filled CMV peaks. We compared peak separation resolution following USP-established equations using 21 CFR part 11 compliant Agilent CDS data analysis software (Agilent Technologies, 2016). We present a robust method to separate AAV8 filled and empty capsids at a high resolution (∼15) using a strong anion exchange HPLC method. The method was tested with multiple experimental runs (N = 15), executed by different analysts (N = 3) in two distinct locations using different qualified instruments with freshly prepared reagent buffers and test aliquots. Our results show robust resolution (>15) and detection of capsid content at broad titer ranges (E+9 to E+13 vp/mL), demonstrating that this method can be broadly used as a reliable quantitative platform to evaluate AAV8 in in-process or QC release samples. We further affirm the separation of empty and filled capsids using orthogonal tools, such as ddPCR, DLS, or ELISA.

## 2 Materials and methods

### 2.1 Empty and filled capsid separation by IEX HPLC

The strong anion exchange separation of the AAV8 empty (VLP) and filled (CMV-GFP) capsids was carried out using an Agilent 1260 Infinity II HPLC system. The system consisted of the quaternary HPLC pump, autosampler, UV detector, fluorescence detector, column oven, and fraction collector. OpenLab 21 CFR part 11 compliant software, version 2.6, was used to control the system and to process all data files. Analytical and QC teams used two different BIA Separations CIMac AAV empty/full strong anion exchange columns to test and separate empty and filled AAV8 capsids. The separation was achieved using a step salt gradient and a Matica custom elution buffer. Mobile phase A was HPLC-grade water, and mobile phase B was custom Matica buffer MD007 (1.0 M tetramethylammonium chloride), mobile phase C was 20 mM magnesium chloride, and mobile phase D was 200 mM Bis-tris propane pH 9.45 (Wang et al., 2019). The step gradient elution method helped resolve the empty and filled AAV8 capsid peaks (Supplementary Figure S1). The method was optimized to elute empty AAV8 capsids at 4.1 min (14% buffer B) and filled AAV8 capsids at 10.1 min (20% buffer B) by modulating the elution buffer B gradient in a stepwise fashion. The peak resolution was calculated using the USP calculation within the Openlabs processing software (Agilent Technologies, 2016). Supplementary Figure S2 explains the resolution calculation.

The method begins with a column equilibration process and ends with a cleaning step. The pump maximum pressure was set to 100 bar, and the volumetric flow rate was 1.30 mL/min. A standard curve was plotted using at least four dilutions of known concentration of AAV8 (filled and empty AAV8 standard curves were plotted separately using commercially acquired AAV8 material). A regression curve was plotted, and the R^2^ of the fit was evaluated by Agilent software (OpenLab v3.5.1 software). The percentage recovery of each standard point was back-calculated after normalizing the calculated concentration to the expected concentration. The relative concentration of test AAV8 empty and filled capsids was determined by comparing the peak areas of an unknown AAV8 peak to known AAV8 standard empty and filled capsids; the calculation was executed using a custom calculation within Openlabs processing software.

### 2.2 Genomic titer determination by droplet digital PCR

Bio-Rad’s ddPCR system was used to quantify the GOI concentration of the AAV8-carrying region of interest. The ddPCR system included an AutoDroplet Generator (Model # 1864101), C1000 Thermo Cycler (Model# 1851197), and QX200 Droplet counter (Model # 1864003). The genomic titers of commercially acquired Virovek AAV8 (Lot # 23-026, Hayward, California) and Virovek AAV8 VLP (Lot # 22-176, Hayward, California) samples, internal AAV8 samples, and HPLC fractionated samples were determined using Matica custom designed primer probes for the known CMV region in the genome.

Prior to testing samples by ddPCR, the samples were treated with DNAse I (0.4 units/mL Clontech DNAse I in 1% Pluronic F-68, 1X Clontech DNAse Buffer, at 37°C for 60 min) to avoid quantifying broken, lysed, or non-encapsulated viral genomes. Samples were then treated with proteinase K (working concentration up to 0.3 mg/mL Qiagen Proteinase K) in Qiagen ProK buffer at 56°C for 60 min to digest capsid proteins, DNAse I, and other proteins in the sample. The treated samples were heated at 95°C for 10 min to denature any remaining capsids and proteinase K. Samples were serially diluted in nuclease-free (NF) water and tested in ddPCR in triplicates. Controls included DNase I and ProK-treated NF water (no template control) and sheared salmon sperm DNA (negative template control).

The master mix for ddPCR was prepared with custom-designed primer probes containing 1X Supermix, 900 nM *CMV* Forward Primer (5′-TGA​CGT​CAA​TGG​GAG​TTT​GTT-3′), 900 nM *CMV* Reverse Primer (5′-ATA​TAG​ACC​TCC​CAC​CGT​ACA​C-3′), 250 nM *CMV* Probe (5′-6-FAM-CATTGACGC-ZEN-AAATGGGCGGTAGG-IABkFQ-3′), and nuclease-free water. Treated sample and controls (6 μL each) were separately mixed with 18 µL master mix in each well at a 1:3 ratio, with a final reaction volume of 24 µL per well. Each sample dilution and control was tested in triplicate. The plate was sealed with Bio-Rad recommended pierceable metal foil using Bio-Rad PX1 PCR Plate Sealer, vortexed for 10 s, and spun down for 30 s using the Thermo Fisher mini-plate spinner. Reaction droplets were generated using the Bio-Rad Automated Droplet Generator on a fresh plate, sealed, and moved onto the thermal cycling step (Bio-Rad C1000 Thermal Cycler). The cycling conditions include maintaining the thermal cycler’s lid at 105°C for the run. Following the master mix and our primer design conditions, the run began with a 10-min enzyme activation step at 95°C, a 30 s denaturation step at 94°C, and a 60 s annealing step at 57°C (Zanker et al., 2022; Prantner and Maar, 2023). The denaturation and annealing steps were sequentially repeated for 40 cycles, after which the reaction was stopped by heating the mixture to 98°C for 10 min. The droplets were held at 4°C until ready for the Bio-Rad QX200 Droplet Reader. The positive and negative FAM droplets were counted and analyzed with a 21CFR part 11 compliant QX Manager Software regulatory edition from Bio-Rad. The threshold for all wells was manually set to 700 based on the negative control wells (no template and negative template control). Using Poisson distribution, the instrument reports results per well in copy/µL. The results were exported and analyzed in Microsoft Excel, and the mean average of sample triplicate (N = 3) was analyzed after compiling the average dilution linearity of samples (N = 3). Results were reported after adjusting for dilutions in copies/mL.

### 2.3 AAV8 physical titer quantification by capsid ELISA

Total AAV8 capsid concentration was determined using a PROGEN AAV8 Xpress ELISA kit (catalog number PRAAV8XP, Heidelberg, Germany). All samples were diluted using the kit-provided assay wash diluent buffer (ASSB1X). The standard curve was plotted using kit-provided AAV8 lyophilized samples at recommended concentrations, and data were fit to a four-parameter logistic curve (4PL Logistic) using a 21 CFR part 11 compliant SoftMax Pro 7.1.2 software. Sample diluent buffer was tested as the negative control plate blank. The assay followed kit recommendations, and the standard curve was used to quantify the results. All dilutions were tested in triplicate, and the background was corrected using the plate blank. The samples were adjusted for the dilution factor, and the average of the triplicate wells was reported.

### 2.4 Size distribution assessment via dynamic and static light scattering

The size and polydispersity of AAV8 samples were quantified using a Wyatt Nanostar DLS II cuvette system (Wyatt; Santa Barbara, CA). Samples were diluted in a known diluent buffer (1X PBS, 0.001% Pluronic F-68) and tested in triplicate at three dilutions. The assay and system suitability control included testing a 30 nm positive size control from Wyatt (Thermo Fisher, Cat# 3030A). Capsids were tested in tandem with static light scattering (SLS) to assess the size distribution, capsid uniformity, and aggregation of particles. Briefly, samples were loaded onto a quartz cuvette (Wyatt; Santa Barbara, CA), and scattering data were recorded for 10 acquisitions per sample and 10 s per acquisition, totaling 100 s of data acquired using 21 CFR part 11 compliant Dynamics 8.1 Software. The process was repeated for three replicates per sample type (n = 30 acquisitions, t = 300 s per sample per dilution). Raw data was exported and analyzed in Microsoft Excel. Analysis criteria were applied to screen for signal quality (signal amplitude ≥ 0.1; baseline within 1.0 ± 0.01). The data was analyzed after compartmentalizing into size bins (regularization ranges: D1: 1–20 nm, D2: 20–50 nm, D3: 50–100 nm, D4: 100–200 nm, and D5: 200–10,000 nm). Because some test samples are impure and have multiple peaks, the frequently occurring peak that is repeatedly identified and measured in more than 15 of 30 acquisitions per dilution (% frequency > 50%) is considered the dominant peak and is analyzed and reported. Population uniformity was determined from percentage polydispersity (%PD) at cumulant data analysis and regularization peak ranges. AAV aggregates or multi-ordered structures were determined from dominant peaks at 3× or larger diameters of expected AAV8 capsid sizes (>75 nm).

## 3 Results

### 3.1 Identity and quantification of AAV8 empty and filled capsids

AAV empty VLP (Lot# 22-176) and AAV CMV-GFP filled capsids (Lot# 22-377) were acquired at 2E+13 vg/mL from Virovek (Hayward, CA). The stock concentrations were provided by vendor certificate of analysis (COA). The AAV8 empty VLP units (in vg/mL) were reported by the vendor using an optical density-based test parameter after QC checks in relation to a known standard curve of AAV8-filled capsids in vg/mL units. Upon executing the customized strong IEX method, the eluted peaks were observed via fluorescence detection (FLD) (fluorescence detector signal of 280 nm at excitation and 350 nm at emission) and UV–vis (260 nm and 280 nm) measurements. Three distinct elution peaks were detected at the 14%, 20%, and 50% elution regions with retention times of 4.1 min, 10.0 min, and 12.5 min, respectively (Supplementary Figure S1). The post-10.0-min peak was lower than the detectable range in other orthogonal methods and was not investigated. No peaks were identified either beyond 18 min at 100% elution conditions or in the follow-up wash steps, indicating all observable peaks were eluted within 15 min and at 75% of the elution buffer (Supplementary Figures S3, S4).

**FIGURE 1:**
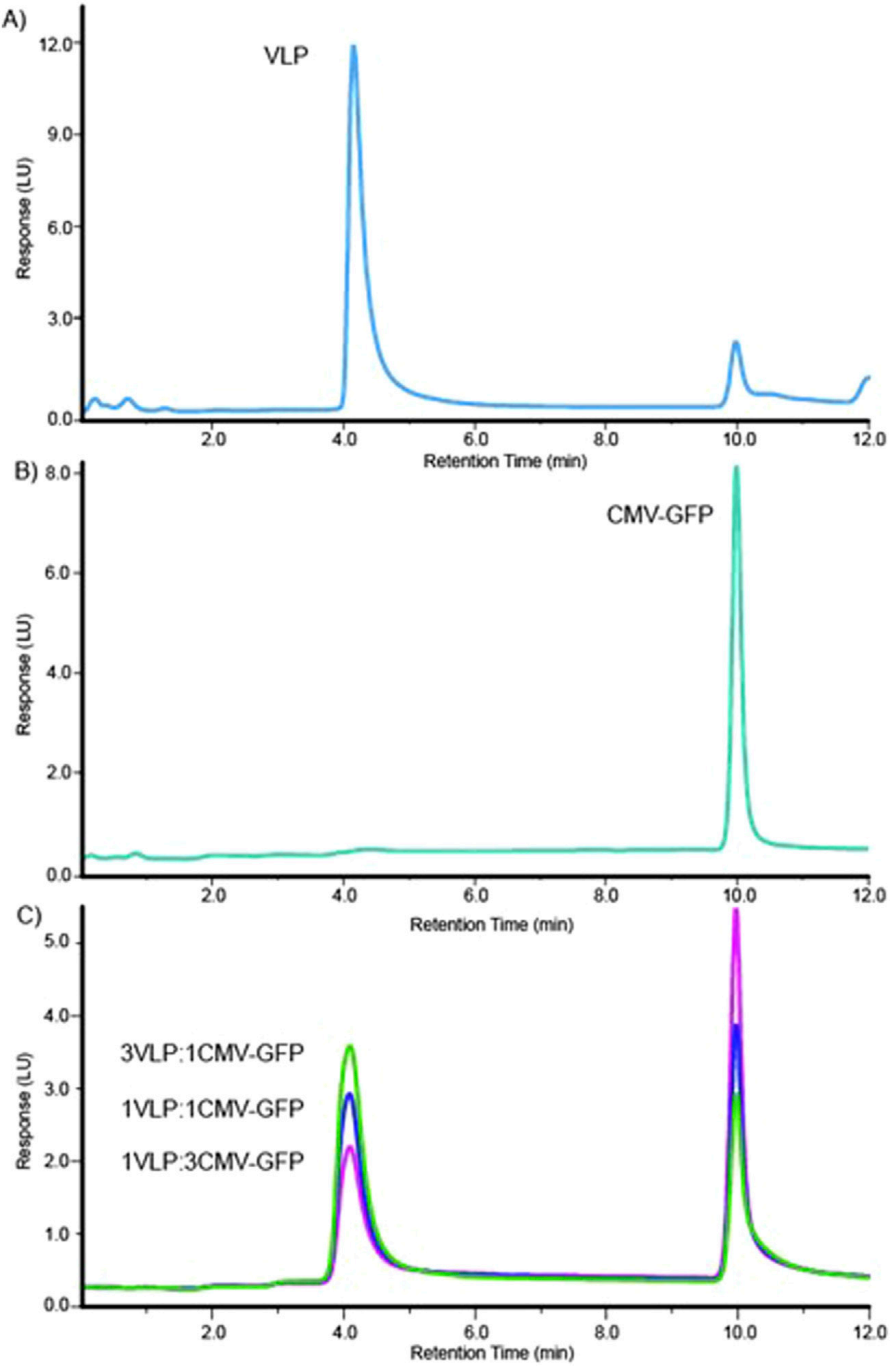
High-resolution method for separation delineates two distinct peaks for AAV8 VLP and CMV-GFP samples. **(A)** The elution profile for the AAV8 VLP sample. **(B)** The elution profile for the AAV8 CMV-GFP sample. **(C)** Overlaid elution profiles of three (3:1, 1:1, 1:3) mixtures of VLP and CMV-GFP.

The sourced stock AAV8 VLP and CMV-GFP capsid samples contained at least two signature peaks that were resolved at the 4.1-min and 10.0-min marks on either sample at different peak areas, as shown in representative characteristic FLD dominant peak of AAV8 VLPs (Figure 1A) and CMV-GFP capsid peaks (Figure 1B). The method resolved the two peaks consistently at a resolution of 15, and the peaks matched respective empty and filled peaks at different weight proportions (% peak area). We determined the 4.1-min peak to be a VLP peak (empty capsid peak) and the 10.0-min peak to be the CMV-GFP peak (filled capsid peak). Measuring the peak area and the 260 nm/280 nm absorbance ratio helped further identify filled and empty capsid peaks. The peaks with a higher 260/280 ratio reflect higher genomic content in filled peaks than empty capsid peaks at similar capsid concentrations. The 10.0-min peak had a larger 260 nm peak area with a 260/280 ratio > 1, while the 4.1-min peak had a smaller 260 nm peak area with a reduced 260/280 ratio < 1 (Table 1). To further test the stability and reproducibility of the high-resolution peak segregation method, a premixed AAV8 sample mixture containing 60% empty and 40% filled capsids was tested multiple times, and the data showed a percentage recovery close to the expected value (the 60% theoretically empty peak was experimentally recovered at 62.1%, and the 40% theoretically filled peak was experimentally recovered at 37.9% recovery). The data were reproducible over N = 7 times (Table 1).

**TABLE 1:** Retention time and resolution.

N = 7	Peak 1 R_t_ (VLP) (min)	% peak 1 area (VLP)	Peak 2 R_t_ (CMV-GFP) (min)	% peak 2 area (CMV-GFP)	Resolution (USP)	VLP 260/280 ratio	CMV-GFP 260/280 ratio
1	4.07	62.7	9.95	37.3	15.56	0.75	1.64
2	4.10	61.0	9.96	38.4	15.38	0.63	1.54
3	4.09	60.8	9.96	39.2	15.66	0.72	1.54
4	4.08	64.5	9.96	35.5	16.58	0.70	1.51
5	4.09	61.9	9.96	38.1	15.77	0.68	1.61
6	4.03	60.5	9.97	39.5	15.86	0.64	1.54
7	4.06	62.5	9.95	37.5	15.32	0.75	1.59
Average	4.07	62.1	9.96	37.9	15.73	0.70	1.57
% CV	0.57	2.2	0.07	3.6	2.67	6.96	2.96

To test the method’s accuracy and precision, we experimentally calculated the peak resolution and tracked the percentage CV of AAV8 samples with known concentrations of premixed empty and filled capsids at three different ratios (1:3, 1:1, 3:1). The method resolved the peaks (>12) and resulted in expected ratios of both percentage VLP and percentage CMV-GFP capsid peak fractions (Table 2). The overlaid chromatograms show changes in the relative percentages of AAV8 VLP and CMV-GFP at three ratios, further supporting each peak’s identity in given samples (Figure 1C). This ensures that the method can detect relative changes in the empty to full ratios within a sample set, which is imperative when testing samples from downstream-filled fraction enrichment operations.

**TABLE 2:** Retention time and resolution of mixed samples.

N = 3 (VLP:GFP)	R_t_ VLP	R_t_ CMV-GFP	% VLP	% CMV-GFP	Resolution	VLP 260/280 ratio	CMV-GFP 260/280 ratio
3:1	4.15	9.98	75.4	24.6	12.7	0.67	1.61
1:1	4.18	9.99	49.5	50.6	12.8	0.66	1.58
1:3	4.16	9.97	25.9	74.1	13.1	0.64	1.57

**FIGURE 2:**
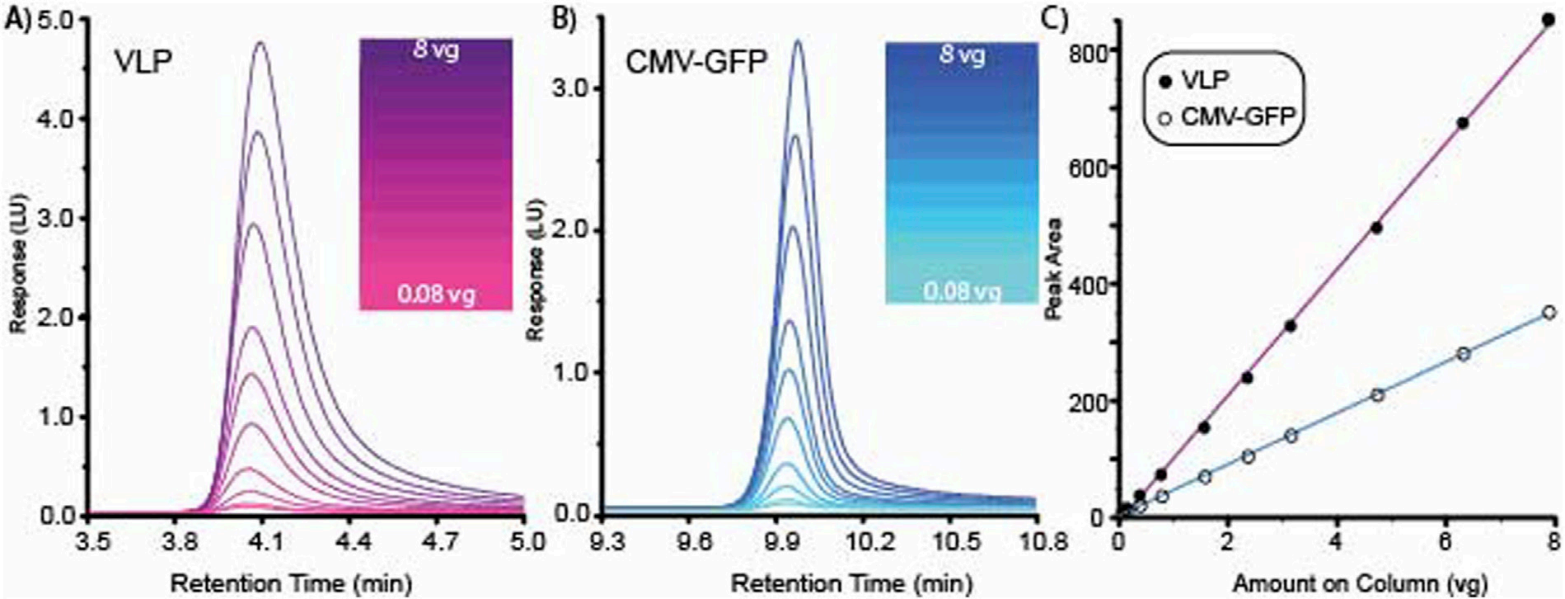
High-resolution method for separating VLP and CMV-GFP demonstrates range and linearity. **(A)** Overlaid chromatogram for 10 AAV8 VLP concentrations. **(B)** Overlaid chromatogram for 10 AAV8 CMV-GFP concentrations. **(C)** Standard curves generated from the 10 concentrations of VLP and CMV-GFP generated graphing peak area vs. amount on the column.

A high-resolution method was tested for range and linearity using multiple AAV8 capsid concentrations. AAV8 VLP empty and CMV-GFP capsids were prepared at a concentration of 1.58E+12 vg/ml. Each empty and filled AAV8 sample was tested at 10 different concentrations ranging from 8.0E+8 to 8.0E+10 vg on column. The overlaid chromatograms for both the AAV8 VLP and the CMV-GFP are shown for VLP empty capsids (Figure 2A) and CMV-GFP filled capsids (Figure 2B). Data were processed using Agilent OpenLabs v3.5.1 processing software, and a standard curve was fitted with a linear model and a 1/X weighting method. The filled AAV8 CMV-GFP capsids’ CMV-GFP peak standard curve had an R^2^ of 0.9995, and the empty AAV8 VLP capsids’ VLP peak standard curve had an R^2^ of 0.99973 (Figure 2C). The average ratio of 260/280 for the 10 AAV8 CMV-GFP capsids injections was 1.59 with a percentage CV of 3.5%, and the ratio of the AAV8 VLP capsid samples was 0.73 with a percentage CV of 4.1%.

### 3.2 Characterization of the IEX-separated AAV8 empty and filled capsids using orthogonal methods

To further characterize empty and filled capsid peaks, the fractions were collected and concentrated using an Amicon Ultra 0.50 mL spin column, frozen at −80°C, and then individually tested for size, polydispersity, and aggregation by DLS, capsid titer by ELISA, and genomic titer by ddPCR (Table 3; Supplementary Figures S5, S6, S7). Three separate aliquots of AAV8 VLP and CMV-GFP capsids were separately tested. The peak fractions at each peak were collected and analyzed. The test included comparing the ratio of total capsid titer by ELISA normalized to genomic titer by ddPCR for each empty and filled peak and sample aggregation and purity by DLS investigations. The capsids were loaded at different concentrations, and the recovered capsid peak titers and peak areas of each peak were normalized to either total capsid titer (% filled specificity ratio) or total peak area (% peak area to total VLP + CMV-GFP peak area). The filled capsid ratio of the VLP fraction (4.10 min collected peak) was less than 1% in both the VLP and CMV-GFP capsid samples (averaging 0.003% in the AAV8 VLP capsids’ VLP fraction and 0.56% in the AAV8 CMV-GFP capsids’ VLP fraction), indicating higher purification and separation of empty capsids at the 4.10-min peak (Table 3). The CMV-GFP capsid fraction (10.0-min peak) for the AAV8 CMV-GFP sample averaged over 40%, indicating selective separation of filled and empty AAV8 capsids. The ddPCR/capsid titer ratio in either peak from each sample shows the amount of genomic titer in the empty AAV8 components is small compared to the full AAV8 capsids based on the number of capsids, illustrating that the method is truly separating the empty from the full AAV8 capsids.

**TABLE 3:** Orthogonal testing results—filled capsid specificity.

Source			ddPCR titer (vg/mL)	ELISA titer (capsid/mL)	% filled ratio (% genomic/capsid titer)	Average % filled ratio
AAV8- VLP capsid	VLP (4.1 min) peak	Source 1	5.48E+06	2.40E+11	0.002	0.003
		Source 2	7.45E+06	3.30E+11	0.002	
		Source 3	9.74E+06	3.00E+11	0.003	
	CMV- GFP (10.0 min) peak	Source 1	3.54E+09	1.10E+11	3.22	1.80
		Source 2	2.26E+09	1.50E+11	1.51	
		Source 3	1.01E+09	1.50E+11	0.67	
AAV8- CMV- GFP capsids	VLP (4.1 min) peak	Source 1	2.93E+08	4.40E+10	0.67	0.56
		Source 2	1.74E+08	3.10E+10	0.56	
		Source 3	1.63E+08	3.60E+10	0.45	
	CMV-GFP (10.0 min) peak	Source 1	1.02E+10	1.80E+10	56.67	45.03
		Source 2	1.48E+10	4.50E+10	32.89	
		Source 3	2.14E+10	4.70E+10	45.53	

Comparing the HPLC peak area for each sample in Table 4 shows that the dominant peaks for each sample source were distinct. The empty VLP capsid sample had a dominant VLP peak area, and the filled CMV-GFP sample had a dominant CMV-GFP peak area (Table 4). Comparing each percentage peak area relative to the total peak area (VLP + CMV-GFP peak area), the VLP fraction of the AAV8 VLP sample and the CMV fraction of the AAV8 CMV-GFP sample had dominant peaks (the AAV8 VLP capsids’ VLP peak ratio was 90.3%, and the filled AAV8 CMV-GFP capsids’ peak ratio was 81%). The unexpected capsid population, the CMV-GFP peak in the empty AAV8 VLP sample, and the VLP peak in the filled AAV8 CMV-GFP capsid sample were less dominant with an average occurrence under 20%, further suggesting that the strong ion exchange method can detect, segregate, resolve, and quantify filled and empty AAV8 populations.

**TABLE 4:** Orthogonal testing results—peak specificity.

Source			HPLC peak area	Average peak area	% peak area/Total peak area
AAV8-VLP capsid	VLP (4.1 min) peak	Source 1	1347.88	1318.49	90.3
		Source 2	1315.87		
		Source 3	1291.73		
	CMV-GFP (10.0 min) peak	Source 1	142.03	141.59	9.7
		Source 2	138.25		
		Source 3	144.49		
AAV8-CMV-GFP capsids	VLP (4.1 min) peak	Source 1	71.73	67.38	19.0
		Source 2	65.69		
		Source 3	64.73		
	CMV-GFP (10.0 min) peak	Source 1	303.4	286.86	81.0
		Source 2	298.31		
		Source 3	258.87		

The DLS light scattering method was used to assess the size distribution and polydispersity of each HPLC fraction peak collected from the source samples. The results (numerical data not shown) showed all fractions exhibited a polydisperse nature (%PD > 30%), with a multimodal size distribution for some sourced fractions. Representative DLS results that support the observation of the numerical data by the presence of multiple peaks are shown in Supplementary Figures S5, S6. The analyzed samples showed three major regularization peak size distributions at D1: 20–50 nm, the AAV8 expected size range (Le et al., 2019; Zoratto et al., 2021), D2: 100–200 nm (higher order aggregate structures), and D3: > 200 nm (larger aggregates and sample impurities). We observed the dominant peak populations are within the expected D1 range for AAV8 capsids (numerical data not shown).

## 4 Discussion

With the advantage of flexible manufacturing, modular functions, and product development with low immunogenicity, AAVs play a crucial role in gene therapy (Naso et al., 2017). While it is known in the CGT industry that AAV production is impacted by partial and empty AAV capsids, along with filled functional capsids (Urabe et al., 2006; Gao et al., 2014; Wright, 2014), current FDA guidelines expect manufacturers to identify and minimize levels of unfilled or partially filled capsids in the final drug product (Arruda, 2021; Sherafat and AC Planning Working Group, 2021). Toward this goal, various techniques have been developed and employed to quantify the amount of filled and empty capsids in in-process AAV samples, including AUC, SEC-MALS, and an orthogonal method using ELISA and qPCR/ddPCR to quantify physical capsid titer and genomic titer, respectively. In addition, the recently emerged charged detection mass spectrometry (CDMS) method has been employed to separate and detect empty, partial-filled, and filled AAV capsids based on their mass/charge ratios (m/z) with promising results (Barnes et al., 2023; Cotham et al., 2024; Jarrold, 2024; McColl-Carboni et al., 2024). However, these techniques, while capable of resolving and analyzing empty vs. filled capsids, have relatively low throughput and require large volumes of pure samples at high concentrations. To compensate for these limitations, the IEX method, which is commonly used in the downstream purification of AAVs, has been applied to quantify the ratio of filled versus empty capsids. Previous work that used the IEX method had tremendous advantages in utilizing different salts, pH buffers, isocratic elution conditions, tailing factors, and column efficiency in different viruses. Nevertheless, to the best of our knowledge, the highest resolution using the IEX technique we have seen from the literature is 7 (Gagnon et al., 2021; Khatwani et al., 2021; Khanal et al., 2023; Kurth et al., 2024). We have observed that a resolution lower than 6 affects in-process sample testing by not giving the analyst sufficient flexibility to demarcate each peak individually, thereby affecting data accuracy. Increased resolution would support testing of a much wider range of concentrations with minimal interference from other impurities affecting data analysis. To accomplish that, the study discussed in this article presents a robust method for the identification and calculation of empty-to-filled AAV8 capsid ratios. The method shown is based on one serotype but can be optimized and applied to other AAVs with minor adjustments to the buffers and elution gradient. The data show excellent peak separation, resolution, precision, and accuracy between the two capsid types. Our modified method uses chloride salts and Mg ions to limit the tailing issues and acquire proper baseline correction. More importantly, the current method successfully identified the retention time and identity of AAV8 VLP and CMV-GFP peaks to be 4.10 min and 10.0 min with a resolution >15 for empty and filled AAV8 capsids, respectively. The method’s selectivity (α), which is the distance of the maxima between two peaks, for the AAV8 VLP and CMV-GFP peaks is 0.41. A selectivity (α) of 1 indicates co-elution of peaks, and we see good selectivity and separation by the optimized method. Column performance (16 × (Rt Peak 1/width Peak1)^2^) was determined to be 273.02. The method was tested with different AAV8 sources, and multiple analysts were trained using at least two different columns and Agilent HPLC instruments at different locations. The method can quantify empty (VLP) and filled (CMV-GFP) AAV8 concentrations in unknown samples using a standard curve of known concentrations of AAV8 VLP and CMV-GFP capsids. The plotted linear–linear fit standard curve with 10 standard points (calculated peak area vs. known capsids loaded onto column) had an R^2^ > 0.99 for both AAV8 VLP and CMV-GFP capsid standard curves. The percentage-CV for each test standard was less than 10%, and the back-calculated percentage recovery of each standard was between 90–110%. The concentration of unknown samples in identical standard buffer conditions can be quantified using the slope and intercept of the standard curve. These features enable the developed strong ion exchange method to support and practice ICH requested guidelines for limit, identity, and quantitative tests (Borman and Elder, 2017).

The study further investigated the segregated peaks with orthogonal methods using UV–vis (260 and 280 nm absorbance measurements and ratio analysis), ddPCR (genomic titer analysis), DLS (size and polydispersity characterization and analysis), and ELISA (total capsid titer analysis). These data clearly show that the method could separate empty and full capsids based on the global charge difference of the capsids. The ratio of empty to filled capsid peak area was reproducible, and the percentage recovery of each peak matched the expected concentration (100%) for known mixtures of VLP and CMV-GFP capsid solutions. The normalized filled specificity value (ratio of genomic titer to capsid titer) was 45% in the AAV8 CMV-GFP fraction in the CMV-GFP peak and less than 1% of CMV-GFP (filled) capsids in the segregated VLP peak. In support of the method applied in testing in-process samples, an example is shown in Supplementary Figure S8.

We recommend that users who adopt this method run a scouting gradient because the effects of temperature and sample storage stability were not determined using this method. Currently, the method is well received internally and is being used to measure the quality of downstream and upstream samples at each stage of development, supporting process optimization.

## Data Availability

The raw data supporting the conclusions of this article will be made available by the authors, without undue reservation.
