# Supplementary material for: An improved high-resolution method for quantitative separation of empty and filled AAV8 capsids by strong anion exchange HPLC
Source: Front Bioeng Biotechnol. 2024 Oct 1;12:1436857. doi: 10.3389/fbioe.2024.1436857 (PMC11473411; doi:10.3389/fbioe.2024.1436857)
Supplement: Supplementary file 1 [file DataSheet1.docx]

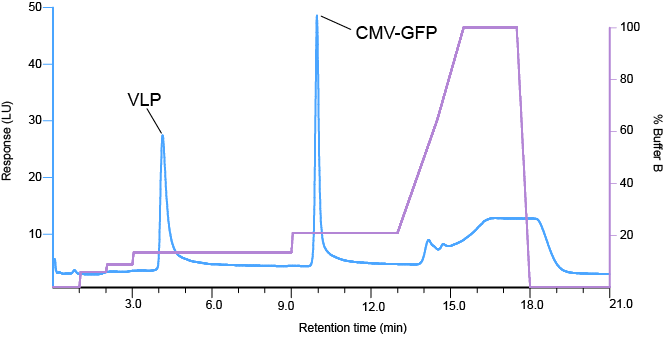


**Supplementary Figure 1:** Chromatographic peaks of mixture of AAV8 VLP and AAV8 CMV-GFP sample observed by FLD. The magenta overlay line is the gradient profile (Right axis) used for the separation. The left axis represents the signal detected by the Fluorescence detector depicted as a response in Luminescence units.


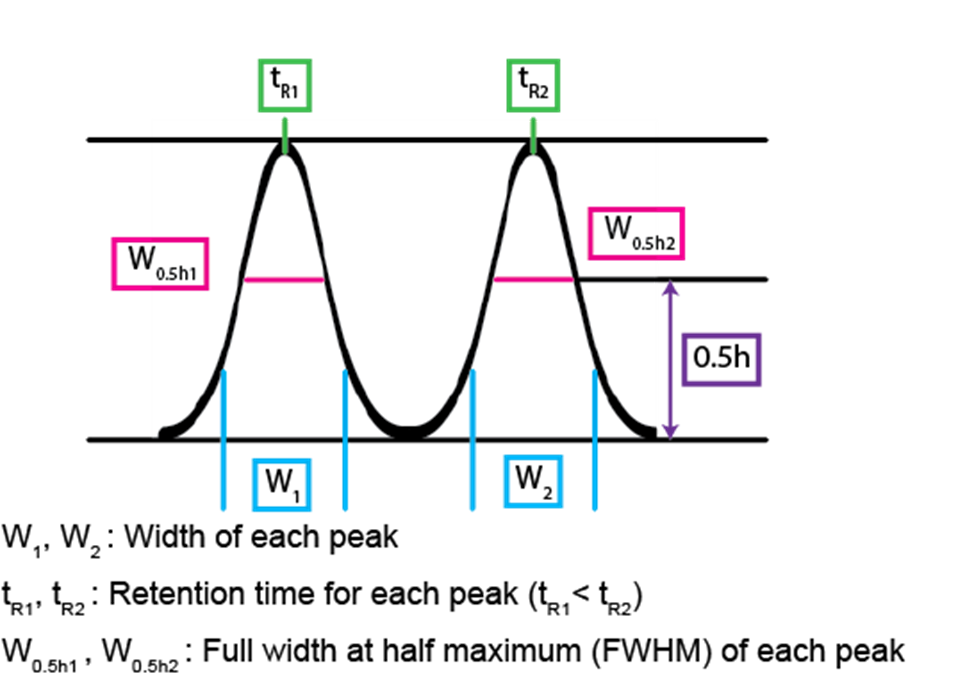


**Peak Resolution = 1.18 (t_R2_ – t_R1_) / (W_0.5h1_ + W_0.5h2_)**

**Supplementary Figure 2:** Peak resolution calculation following USP (United States Pharmacopeia) characteristics. Firstly, the baseline needs to be identified, and individual component peak marked and named. The agilent OpenLab software identifies individual peak width (W1 and W2) and peak full width at half maximum (W_0.5h1_ and W_0.5h2_ FWHM) in assigned units. Peak resolution is calculated between the identified component peaks through the 21 CFR part 11 compliant software.


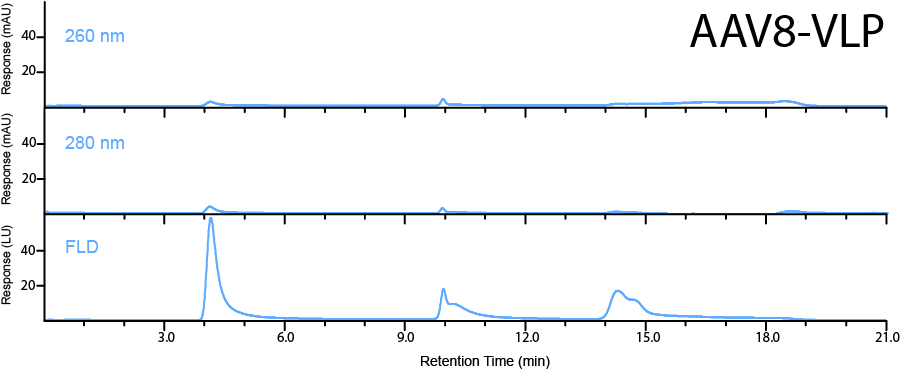


**Supplementary Figure 3:** Chromatographic peaks observed by UV 260 and 280, and FLD traces with AAV8-VLP sample. Peaks detected at 4 and 10 minutes were further investigated by orthogonal methods. The peak at 14.5 minutes was undetectable by orthogonal methods and not investigated. No signal was observed beyond 18 minutes.


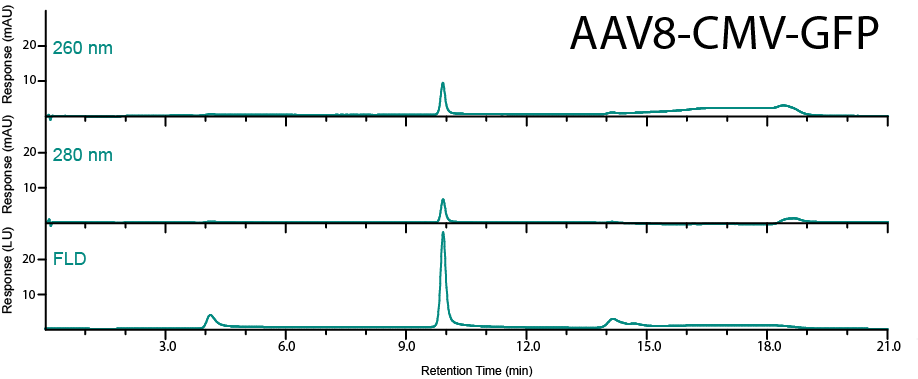


**Supplementary Figure 4:** Chromatographic peaks of AAV8 CMV-GFP sample observed by UV 260 and 280, and FLD traces when injecting the Virovek CMV-GFP only. The 260/280 ratio for the CMV-GFP can clearly be seen on the UV traces.


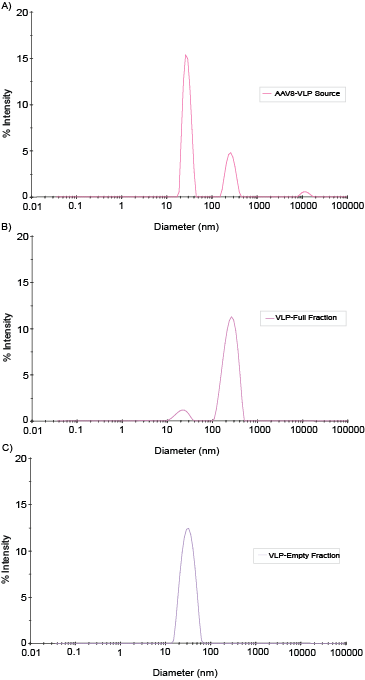


**Supplementary Figure 5:** Representative light scattering (DLS) results for AAV8 VLP Sample is shown. Each curve is an overlay of over 10 data acquisitions acquired for 30 seconds each. **(A)** The hydrodynamic diameter light scattering results of source (untreated) AAV8 VLP samples is shown. **(B-C)** After fractionating AAV8-VLP source sample under our custom IEX method, the acquired AAV8 CMV GFP fraction peak **(B)** and AAV8 VLP peak **(C)** were isolated and DLS hydrodynamic diameter results are presented.

 
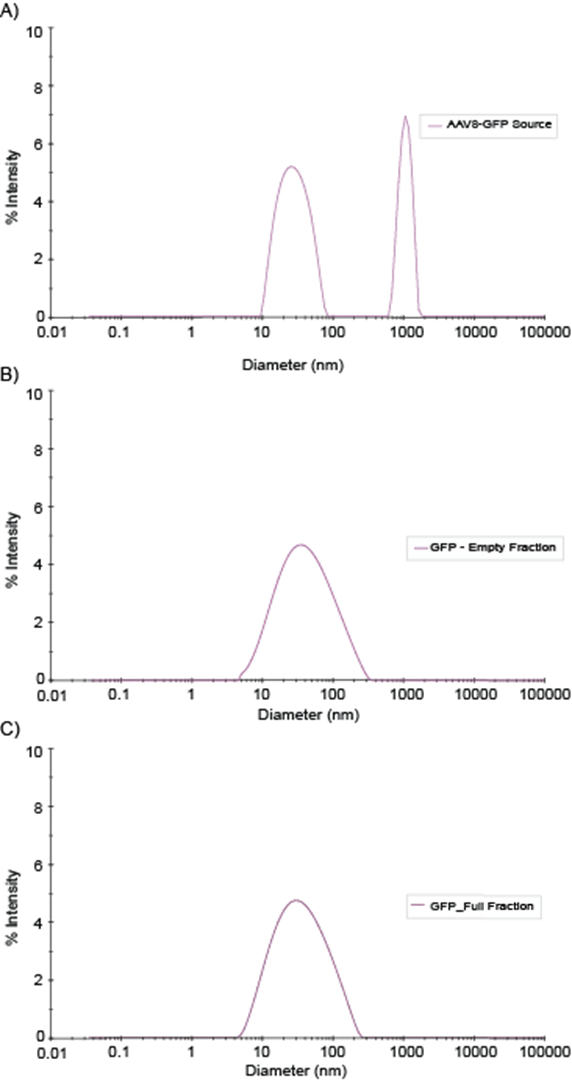


**Supplementary Figure 6:** Representative light scattering (DLS) results for AAV8 CMV-GFP Sample is shown. Each curve is an overlay of over 10 data acquisitions acquired for 30 seconds each. **(A)** The hydrodynamic diameter light scattering results of source (untreated) AAV8 CMV-GFP samples is shown. **(B-C)** After fractionating AAV8-CMV-GFP source sample under our custom IEX method, the acquired peaks were isolated and tested. The AAV8 VLP peak fraction or “GFP empty fraction**” (B)** and AAV8 CMV-GFP peak fraction or “GFP Full fraction” **(C)** were isolated and DLS hydrodynamic diameter results are presented.


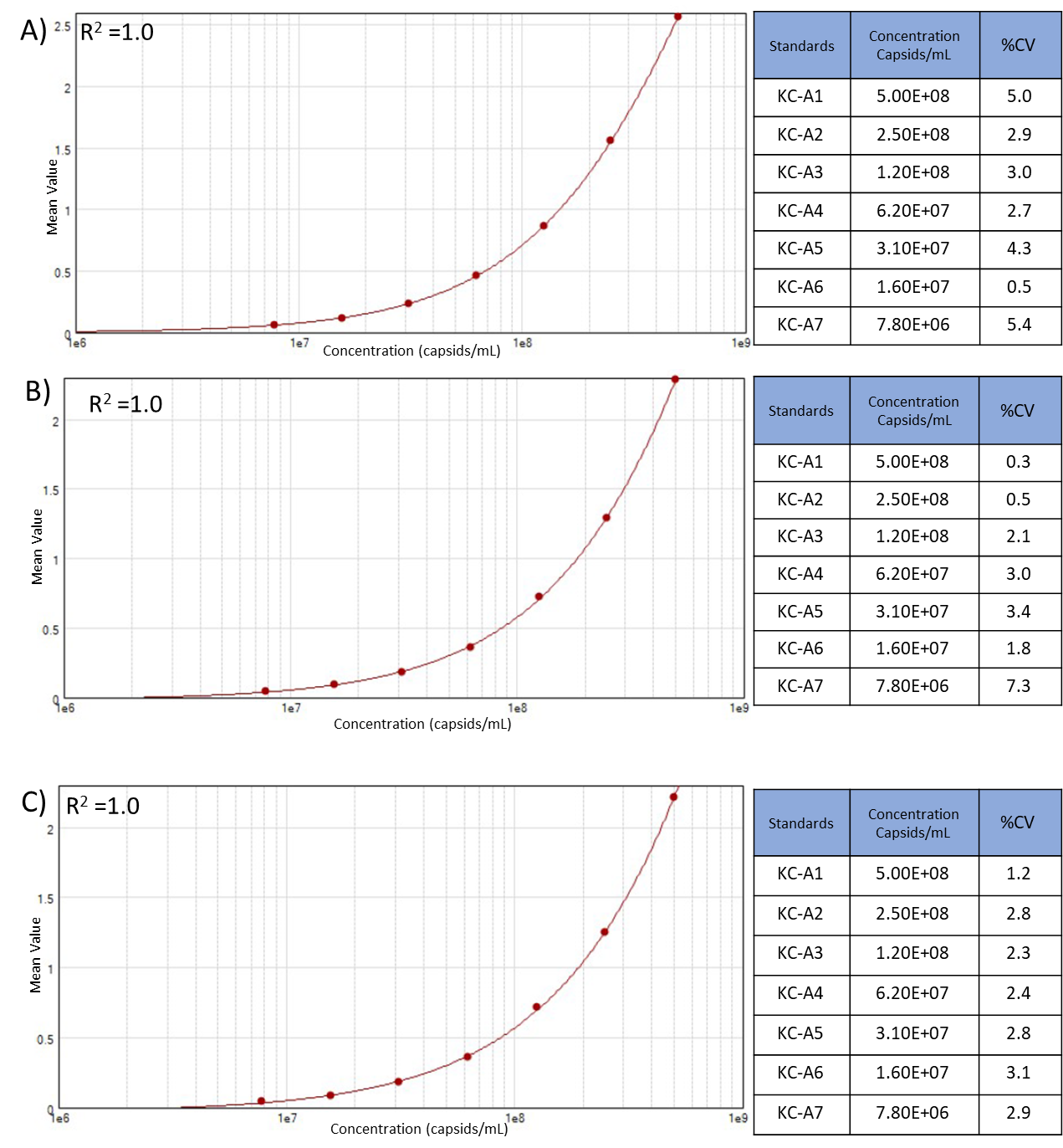


**Supplementary Figure 7:** ELISA standard curve from known concentration of kit provided standards are shown. Standard data points were fit to a 4-PL curve as recommended in the kit manual, and unknown sample concentrations were indirectly evaluated in capsids/mL from the standard curve.


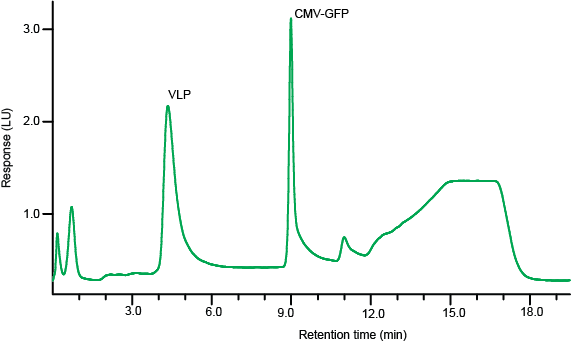


**Supplementary Figure 8:** A representative result from an in-process sample analyzed under our custom IEX-HPLC method is shown. The method can be used to quantify AAV8 empty: full peak ratio for samples and quantify relative AAV8 VLP and AAV8 CMV-GFP concentrations using a known standard curve.
